# Blood in Its Relation to Surgical Diagnosis1Read before section on surgery, Buffalo Academy of Medicine, February 4, 1902.

**Published:** 1902-10

**Authors:** A. E. Woehnert

**Affiliations:** Buffalo, N. Y., 155 Allen Street.


### Blood in Its Relation to Surgical Diagnosis.[1]

By A. E. WOEHNERT, M. D., Buffalo, N. Y.

THE question before us is what value, if any, to the surgeon is the examination of blood? Will any of the data obtained by such analysis aid him in the diagnosis or treatment of his patient?

It has seemed to me that the surgical part of the profession here in Buffalo was rather lukewarm in regard to the newer diagnostic methods. Judging from the articles one sees in the journals, this is true elsewhere. An article appeared in one of the leading journals some months ago, based upon the examination of blood in one case of appendicitis, condemning such examination on account of failure in this particular instance. I am not going to try and convince you that the results obtained by the examinations are infallible, but that in a great majority of cases the blood can be relied upon to give information either in a positive or negative way.

The analysis of the blood by modern methods includes the estimation of the hemoglobin, the determination of the specific gravity, and the count of the erythrocytes and leucocytes. The general morphology of the red cells and the differential counts of the leucocytes are obtained by the stained specimen. The rough estimation of the fibrin present with estimation of coagulation time should be made in proper cases. The hemoglobin, estimated by one of the colormetric methods or by means of specific gravity, is of value in a prognostic sense in surgery. All things being equal, the patient whose hemoglobin is near the normal point has a better chance to withstand the shock of a severe operation than one whose iron is markedly deficient. Operative measures of any kind become dangerous in patients whose hemoglobin is much below 50 per cent., and where possible should be postponed and therapeutic measures employed to bring the coloring matter near the normal standard.

1. Read before section on surgery, Buffalo Academy of Medicine, February 4, 1902.

I have seen two cases of pernicious anemia within the year who narrowly escaped operation for cholelithiasis on account of the lemon tint characteristic of this form of anemia. A simple estimation of the hemoglobin in such a case would have made this mistake impossible.

The Justice test is of value in suspected syphilis, and depends upon the diminution of the hemoglobin in specific cases when mercury is administered. There may be a fall in twenty-four hours from 10 per cent. to 15 per cent. in the syphilitic cases, whereas in the non-syphilitic cases no such fall is observed. In the matter of shock, the estimation of hemoglobin is of value in separating the nervous form from that due to hemorrhage.

The administration of iron in all cases should be governed by one of the instruments of precision. Many times an anemia apparently exists when none is present. The red cells of the blood are of some importance to the surgeon. They are maintained at near the five million mark in the normal individual. There are slight variations in number throughout the day, but not more than two or three hundred thousand to the cubic millimeter. Any great variation would point to serious constitutional disturbance. Roughly speaking, when the red cells are near the one million mark in number, pernicious anemia is usually the underlying cause; between one and two million, we might have pernicious anemia, cancer, leukemia, chlorosis, and extreme secondary anemia, as the cause. The reduction of the reds to three or four million may be due to a large variety of causes, including chlorosis, pernicious anemia, leukemia, and the secondary anemias due to many causes.

The study of variations in the general appearance of the erythrocytes, as seen in the stained specimen, is also of value. The normal cells differ very slightly in their size and shape. The essential anemias are characterised by great variations in this regard. The general picture of a stained specimen of pernicious anemia is that of increased size of individual cell or macrocytosis. Poikilocytosis or irregularity of contour is also characteristic of this form of anemia. Chlorosis is characterised by small cells or microcytosis. In both diseases large and small cells may be found. The secondary anemias, on the other hand, even where great reduction in the number of cells is found, are not so apt to show this peculiarity. Nucleated cells are commonly found in severe oligocythemia from any cause, and their prevalence is an index to a certain extent of the demands made upon the blood-making organs of the individual. The severer the process at work, the more nucleated cells found and the more embryonic they become in type. The large nucleated cells or gigantoblasts are typical of pernicious anemia.

Many times the reds may take not only an acid, but alkaline stain as well. Very often the protoplasm is filled with small blue granules. This polychromataphilia and granular degeneration is found in toxic conditions of the blood. In these particulars the study of the red cells may yield information of value, often being diagnostic or pointing to an underlying cachexia or toxemia. They always give an index to the general condition of the patient under observation.

The study of the number and kind of leucocytes is the most interesting and important part of the blood examination to the surgeon. The count should be made with the special pipette. Toison's solution and red counter often giving misleading results on account of high dilution unless the special counter chamber is used. The blood should be taken at a stated time, as long after a meal as possible in order to do away with the digestive leucocytosis. A simple count is not sufficient and may lead to erroneous interpretation. I have often found a leucocytosis of 14,000 to 15,000 in cases where pus was suspected, but the increase was in the young cells or lymphocytes. A differential count is necessary in order to arrive at a correct result. The estimation of the leucocytosis, made by examining the fresh blood, is proper as a preliminary test or where differentiation from malaria is desired. We should not base an opinion on such a procedure alone. Most failures in the blood as a diagnostic factor can be attributed to incomplete analysis and slipshod methods. In important cases a single examination is not sufficient any more than a single urinary examination, and positive results should not be expected from such examinations.

The history is often of great importance to the hemotologist in order that he may arrive at a correct conclusion. The blood analysis, like that of the urine, can only be counted as a single item in summing up a given case. The leucocytes in the normal blood number about 7,500 to the cubic millimeter, and are made up of four varieties of cells, namely:

| | |
|---|---|
| Polymorphous or adult cells . . . . . . | 66 per cent. |
| Small lymphocytes . . . . . . . . . . | 26 " " |
| Large lymphocytes . . . . . . . . . . | 6 " " |
| Eosinophiles . . . . . . . . . . . . . . | 1 " " |

Physiologically, we have increase of leucocytes after meals, during pregnancy, after hemorrhage and after a bath. Pathologically, the leucocytes are increased in a variety of conditions. We have a leucocytosis in many non-surgical infections —in pneumonia, which may serve as a type, the leucocytes may reach twenty or thirty thousand.

In the pyogenic infections, blood count is often invaluable. In all pus infection we have a leucocytosis of a polymorphous variety. There are certain rare cases where the pus has been thoroughly walled off, or where, through overwhelming numbers or virulence of germ, no reaction occurs. One such case has recently come under my observation. A patient after confinement had remittent temperature for some weeks with loss of weight and strength, with occasional sweats. The blood analysis gave 3,280,000 reds; leucocytes, 10,600; polymorphous cells, 85 per cent.; hemoglobin, 50 per cent.; fibrin not increased. In spite of the blood showing no leucocytosis, several ounces of pus were found on operation.

A second case—a woman who had been treated a year for malaria. The blood analysis gave the following results: leucocytes, 23,800; polymorphous, 84 per cent.; hemoglobin, 60 per cent. This case had been a very puzzling one, and after blood examination a renewed search was made; pus was finally found in the pelvis.

In a number of appendix cases reported last year in which blood analysis was made, when the leucocytes numbered 15,000 or over, pus was found. Increase in the number of leucocytes meant a spread of the process, while a drop in leucocytes heralded an improvement in the case. The progress of a pyogenic infection, especially in the appendix, can be followed with accuracy by a daily leucocyte count.

The blood of cancer where cachexia is present often shows a leucocytosis, even where secondary infection or ulceration has not occurred. This may be brought about by the cancer organism. The leucocytes may reach a considerable figure. The study of the erythrocytes, which show considerable change in size and shape and the presence of nucleated cells, will help to differentiate. The fact that fibrin is decreased rather than increased is also of value to differentiate from pyogenic process. Moreover, the increase is often in lymphocytes rather than the polymorphous leucocytes. The absence of leucocytosis is as valuable and conclusive as its presence in many instances.

Occasionally one comes in contact with cases which are puzzling and may be one of several conditions where treatment would radically differ. The presence or absence of a leucocytosis may settle the nature of the case. As an example, a year ago I saw a little girl who had had some pain in the region of the appendix for the previous ten days. When seen by me, she had a temperature of 102°; pulse, 120, was chilly, and there was tenderness and rigidity over McBurney's point. She had some coryza and conjunctivitis. Whether this temperature and pulse were due to appendicitis or oncoming measles, I could not say. A blood examination was made. The leucocytes numbered 6,000. Thirty-six hours later she had a well-developed measle eruption. The tenderness over the appendix was present for some days however.

We occasionally run across obscure abdominal cases, ofttimes ushered in with a chill or chilly sensations. These cases are perplexing. It is important to know whether we have to deal with typhoid, appendicitis, or some other inflammatory abdominal condition. Here, blood analysis is invaluable. The presence of a Widal reaction is diagnostic of typhoid. A polymorphous leucocytosis speaks for appendix or other inflammatory process. The presence of an eosinophilic leucocytosis may be diagnostic of trichinosis. In the presence of a splenomegaly, the blood is also of value. Leukemia is easily detected by the enormous increase in leucocytes, the majority of which are the characteristic myelocytes. The presence of polymorphous leucocytosis with characteristic changes in the reds and fibrin decrease may mean cancer. Where fibrin is increased, abscess of the spleen or embolism from aseptic endocarditis may be suspected. Absence of leucocytes where the plasmodium is not found points to splenic anemia.

Thus the blood in the present state of our knowledge may be of considerable value in differential diagnosis. In the course of time we may be able to test the quality of the serum and arrive at an exact knowledge of its composition. We may have serum tests for the commoner infections and be able to supply the various antitoxic serums necessary to combat them. At the present time we can, with justice, say that the study of the blood is important and valuable as an aid to differential diagnosis. Not always positive in its conclusions and often diagnostic in a negative way.

155 ALLEN STREET.

